# Selected Ion Flow Tube-Mass Spectrometry (SIFT-MS) as an Alternative to Gas Chromatography/Mass Spectrometry (GC/MS) for the Analysis of Cyclohexanone and Cyclohexanol in Plasma

**DOI:** 10.1021/acsomega.1c03827

**Published:** 2021-11-19

**Authors:** Colin Hastie, Adrian Thompson, Mark Perkins, Vaughan S. Langford, Michael Eddleston, Natalie ZM. Homer

**Affiliations:** † Anatune Ltd, Unit 4, Wellbrook Court, Girton Road, Cambridge CB3 0NA, United Kingdom; ‡ University/BHF Centre for Cardiovascular Sciences, Queen’s Medical Research Institute, 47 Little France Crescent, Edinburgh EH16 4TJ, United Kingdom; § Syft Technologies, 68 St Asaph St, Christchurch 8011, New Zealand; ∥ Mass Spectrometry Core, Edinburgh Clinical Research Facility, University/BHF Centre for Cardiovascular Sciences, Queen’s Medical Research Institute, 47 Little France Crescent, Edinburgh EH16 4TJ, United Kingdom

## Abstract

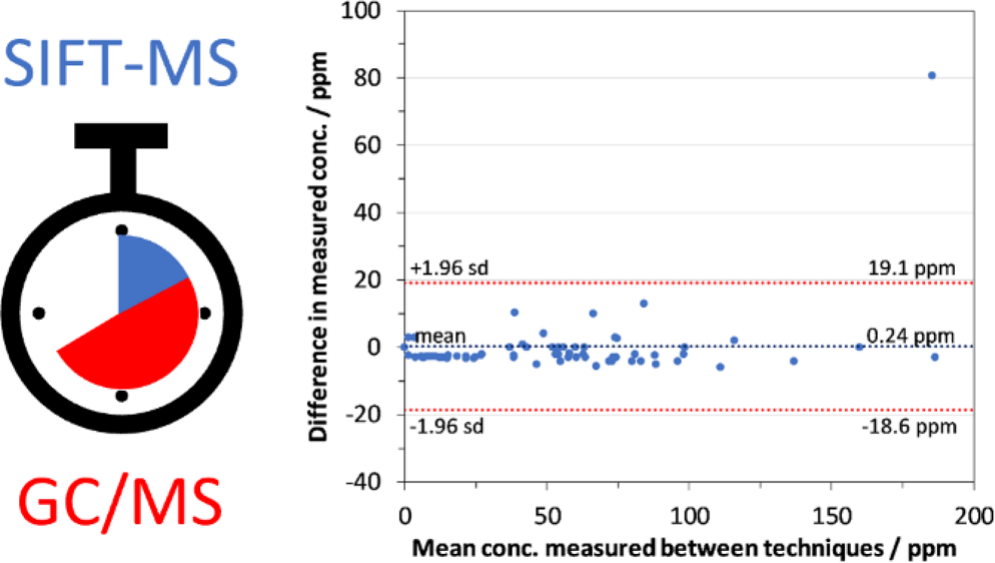

Self-poisoning with professional agricultural pesticide products is responsible for about 20% of global suicide, with most cases occurring in South Asia and China. Treatment of severe poisoning involves long-term intensive clinical care and is often unsuccessful. Solvent co-formulants (such as cyclohexanone) also contribute to mortality themselves or via more toxic metabolic products (such as cyclohexanol). Faster detection of co-formulants could aid earlier identification of pesticide poisoning and faster intervention, reducing mortality. Conventional analysis of volatiles in blood uses headspace (HS)-GC/MS. This paper evaluates SIFT-MS, a direct MS technique that provides higher sample throughput than GC/MS, as a potential tool for cyclohexanone and cyclohexanol analysis in plasma. Both instruments were calibrated using a conventional approach prior to analysis of each porcine plasma sample on both instruments. Comparative data were evaluated using Bland–Altman plots, demonstrating that the techniques were in good agreement. Compared with GC/MS, SIFT-MS provides fourfold higher sample throughput and shows great promise as an alternative analytical tool.

## Introduction

Self-poisoning with professional agricultural pesticide products is responsible for about 20% of global suicide, with most cases occurring in South Asia and China.^1,2^ Treatment of severe poisoning involves long-term intensive clinical care and is often unsuccessful.^3−5^ Harm following ingestion is caused not only by the pesticide itself but also, in some cases, by solvent co-formulants such as cyclohexanone.^6,7^ The samples reported here were collected from an ongoing preclinical study to investigate the effects of organophosphorus pesticides and the solvent cyclohexanone (which is metabolized to the more toxic cyclohexanol) on neuromuscular function in terminally anaesthetized pigs. To understand the concentration–effect relationship, we sought to measure the concentration of both cyclohexanol and cyclohexanone in plasma.

Current gold standard targeted analytical methods for simultaneous analysis of small molecules and their metabolites, such as steroids, use mass spectrometry coupled to liquid or gas chromatography.^8^ The detection of volatile substances such as ethanol^9^ and isoflurane^10^ in blood is enabled by headspace-gas chromatography/mass spectrometry (HS-GC/MS), and this approach can be applied to the measurement of volatile cyclohexanone and its metabolite cyclohexanol. However, HS-GC/MS analysis is costly and time consuming. Faster analysis is most conveniently achieved using direct analysis methods that eliminate the slower temporal separation of chromatography but that are connected to a rapid, selective mass spectrometric (MS) method. Prominent among these methods is the more recent soft chemical ionization method selected ion flow tube-mass spectrometry (SIFT-MS).^11,12^

SIFT-MS applies highly controlled soft chemical ionization coupled with mass spectrometric detection to rapidly quantify VOCs to part-per-trillion concentrations by volume (pptV).^13^ The most advanced instruments have eight chemical ionization agents (reagent ions): H_3_O^+^, NO^+^, O_2_^+^, O^–^, O_2_^–^, OH^–^, NO_2_^–^, and NO_3_^–^.^14,15^ These reagent ions react with VOCs, but they do not react with the major components of air (N_2_, O_2_, and Ar) and only slowly with water, enabling analysis to be conducted without the need for pre-concentration, derivatization, or drying of samples.

The SIFT-MS reagent ions are also rapidly switchable using a built-in quadrupole mass filter, providing high selectivity in the absence of chromatographic pre-separation or high-resolution mass spectrometric detection. When automated, SIFT-MS provides higher sample throughputs^16^ and is much simpler to operate than equivalent GC/MS instruments. As such, SIFT-MS also has potential for application in clinical settings. However, it has yet to be validated for analysis of volatile organic compounds (VOCs), such as cyclohexanone and cyclohexanol (Table 1), in plasma. Although SIFT-MS has previously been shown to compare well with an accepted chromatographic method for environmental VOC analysis,^17^ the present study represents the first comparison of SIFT-MS with gold-standard HS-GC/MS for analysis of plasma. This study aimed to compare SIFT-MS with GC/MS for headspace analysis of cyclohexanone and cyclohexanol in porcine plasma samples.

**Table 1:** 

compound	CAS number	SMILES^a^	boiling point (°C)	Log *K*_ow_	vapor pressure (mm Hg)	Henry’s law (atm m^3^/mole)
cyclohexanone	108-94-1	C1CCC(=O)CC1	155.4	0.81	5.0	9.00 × 10^–6^
cyclohexanol	108-93-0	C1CCC(CC1)O	160.8	1.23	5.17	4.40 × 10^–6^

a SMILES: simplified molecular input line entry system.

## Results and Discussion

### Method Performance

**Figure 1:**
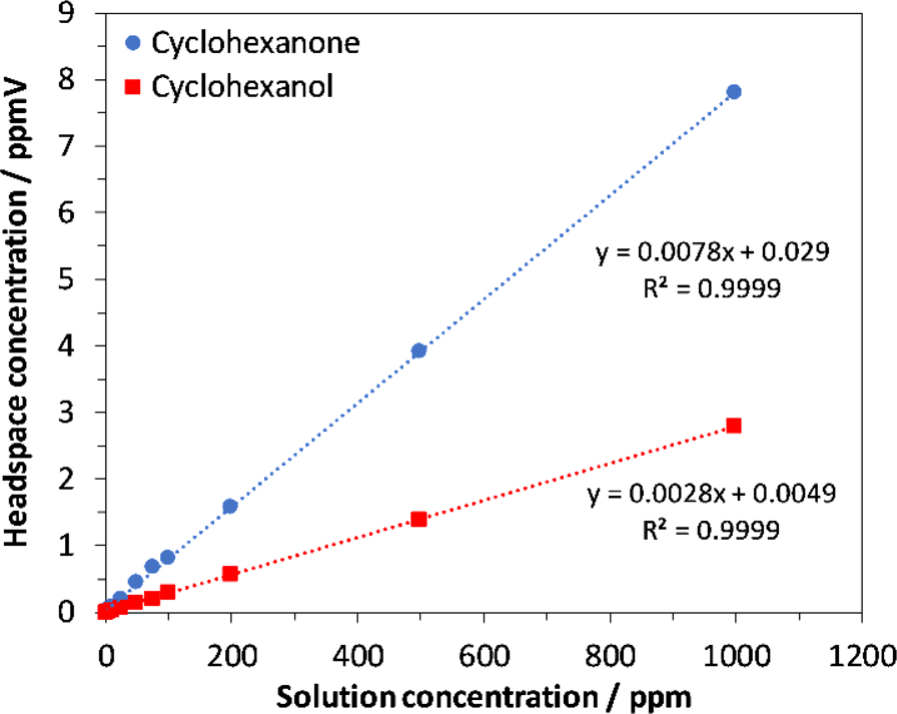
Calibration plot of cyclohexanone and cyclohexanol standards analyzed by SIFT-MS.

**Figure 2:**
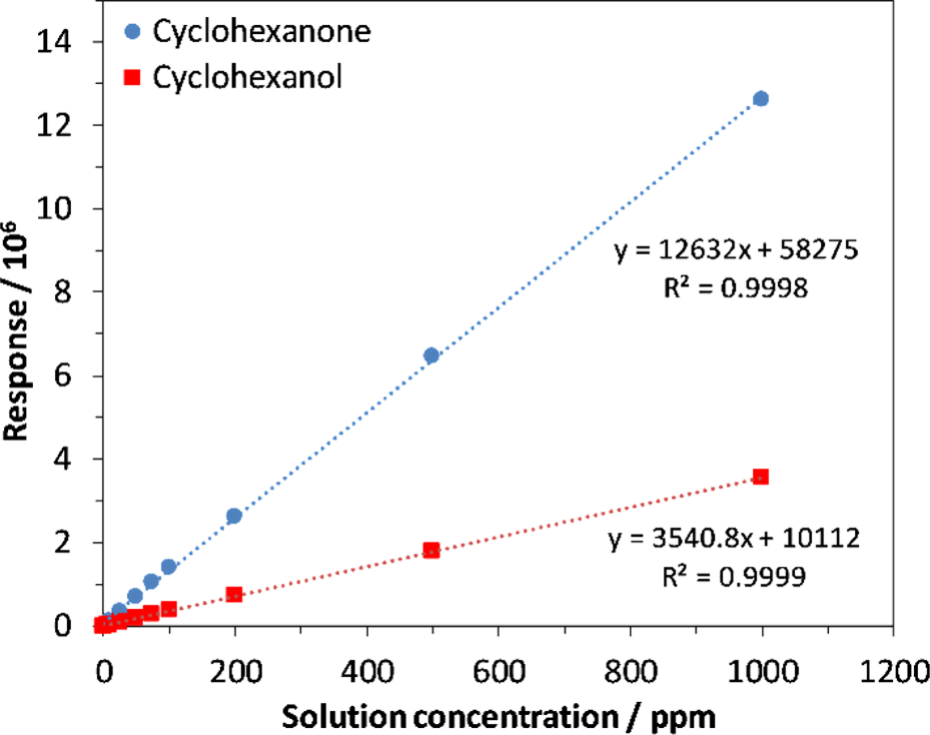
Calibration plot of cyclohexanone and cyclohexanol standards analyzed by GC/MS.

Performance of the analytical methods was determined by assessing the linearity and the reproducibility of calibration standards. Following linear regression analysis, GC/MS was calibrated (2–1000 ng/L) with *r*^2^ values of 0.9998 and 0.9999 for cyclohexanone and cyclohexanol, respectively. SIFT-MS calibration plots had *r*^2^ values of 0.9999 and 0.9999 for cyclohexanone and cyclohexanol, respectively. Calibration plots are shown for SIFT-MS (Figure 1) and for GC/MS (Figure 2). A standard at a concentration of 200.0 ppm was analyzed throughout the sample analysis (*n* = 9). By GC/MS analysis, average concentrations of 200.0 ± 8.8 and 213.0 ± 19.9 ppm were measured for cyclohexanone and cyclohexanol, respectively. By SIFT-MS, average concentrations of 193.0 ± 7.4 and 203.0 ± 9.3 ppm for cyclohexanone and cyclohexanol, respectively, were measured.

### Sample Analysis

**Figure 3:**
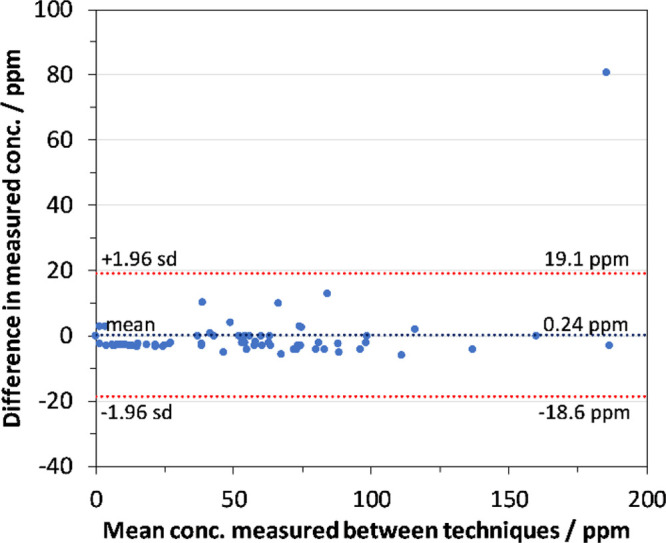
Bland–Altman plot comparing measured concentration of cyclohexanone determined by GC/MS and SIFT-MS in the same plasma samples.

**Figure 4:**
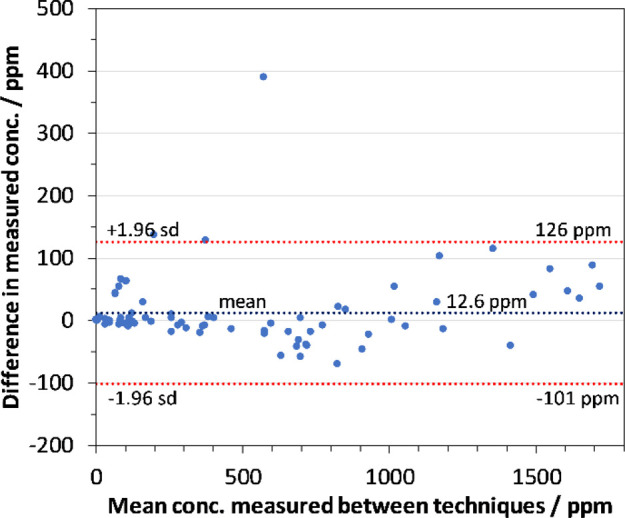
Bland–Altman plot comparing measured concentration of cyclohexanol determined by GC/MS and SIFT-MS in the same plasma samples.

The same plasma samples were analyzed using both analytical methods, GC/MS and SIFT-MS. Samples were quantified against calibration curves for each analytical method and the resulting concentrations for the two techniques were compared. Figures 3 and 4 compare the results for cyclohexanone and cyclohexanol, respectively, for both analytical methods using Bland–Altman plots.^21^ A near *x* = *y* correlation in the measured concentration by each technique can be seen. One outlier was noted, which is believed to be the result of a leak in the headspace vial, because it was noted after analysis that there was damage to the rim of the vial. As SIFT-MS analysis was conducted after GC/MS analysis, this would result in a lower concentration by SIFT-MS. An additional pig sample had particularly high levels, measuring concentrations of 3120 and 3920 ppm for cyclohexanol by SIFT-MS and GC/MS, respectively. However, as these concentrations are significantly above the calibrated range, they have not been included in the figures.

### Consideration of Choice of Techniques

Both techniques have their merits. SIFT-MS allows greater throughput of analysis. For this method, 70 samples were analyzed in 6 h by SIFT-MS compared to 24 h for the same number of samples by GC/MS (a fourfold increase). Due to the chromatographic separation, GC/MS provides slightly greater selectivity in analyte identification, whereas in SIFT-MS, multiple reagent ions provide specificity.

Chromatographic separation ahead of mass spectrometry analysis can address ion suppression and ion attenuation in clinical analysis.^22^ However, because SIFT-MS is a direct-injection chemical ionization technique, ionization only converts a small percentage of the reagent ion to product ion so it avoids issues with ion suppression and ion attenuation seen in some other MS techniques. There is no need for separation. As long as the total analyte concentration falls within the linear range (Figure 3), then linear and separable analysis of trace analytes is seen.

Appropriate blank analysis would identify any potential interference present that could affect specificity. Both methods showed a linear response and sufficient sensitivity to quantify analytes at clinically relevant concentrations in this preclinical model. It was noted that some carryover was observed after analysis of samples with high levels. The level of carryover was half as significant in the SIFT-MS analysis compared to that of GC/MS, likely due to smaller surface areas in the inlet of the SIFT-MS instrument.

## Conclusions

Headspace GC/MS and SIFT-MS were successfully applied to analysis of cyclohexanol and cyclohexane (Table 1) in pig plasma. External standard calibration showed both techniques to give linear and reproducible results. Plasma sample concentrations ranged from <2 to 226.0 ppm cyclohexanone and <2 to 3920 ppm cyclohexanol. The measured concentration by each technique showed comparable results on almost all samples, with the only significant deviation in measured concentration between analysis methods believed to be due to legitimate outliers arising from sample damage. The relatively high throughput that automated SIFT-MS allows makes it an appealing technique for rapid analysis of plasma samples for the cyclohexanone co-formulant in pesticides and its metabolite cyclohexanol.

## Experimental Section

### Materials and Reagents

Analytical standards of cyclohexanol and cyclohexanone were purchased from Sigma-Aldrich (Dorset, U.K.). Saline solutions (0.1 M) were prepared using MilliQ ultrapure water (Watford, U.K.) and sodium chloride was from Sigma-Aldrich (Dorset, U.K.).

### Sample Preparation

The preclinical mini-pig model utilized commercial breed male pigs weighing approximately 15 kg, which were housed under standard conditions. All procedures were performed under the aegis of the U.K. Animals (Scientific Procedures) Act, 1986, and the EU directive 2010/63/EU and with the local ethical committee approval. On the day of the study, they were anaesthetized and instrumented as described previously.^18^

Cyclohexanone and cyclohexanol were administered as follows. Cyclohexanone (>99.5%) and cyclohexanol (99%) were prepared as 5 and 3.5% solutions (v/v), respectively, in sterile physiological saline immediately before administration. These solutions were infused intravenously over 30 min periods. Infusion volumes were calculated to give between 125 and 500 mg/kg bodyweight of the compounds according to the study design. Multiple infusions were given to each animal.

Blood samples were collected from pigs into K_2_EDTA coated tubes and centrifuged (2500*g*, 4 °C for 5 min). Plasma was aliquoted into 2 mL vials in duplicate and stored at −80 °C prior to analysis by two analytical techniques.

Plasma samples were prepared for MS analysis by pipetting 1 mL into 20 mL headspace vials and storing at −20 °C prior to analysis. The concentration of cyclohexanone and cyclohexanol were determined in the samples by both analytical methods. The analytes were quantified against calibration standards prepared in 0.1 M sodium chloride solution in MilliQ ultrapure water, over the range of 2–1000 ppm cyclohexanol and cyclohexanone. Samples and standards were heated for 15 min at 40 °C in an agitator prior to injection.

### Headspace GC/MS Analysis

**Figure 5:**
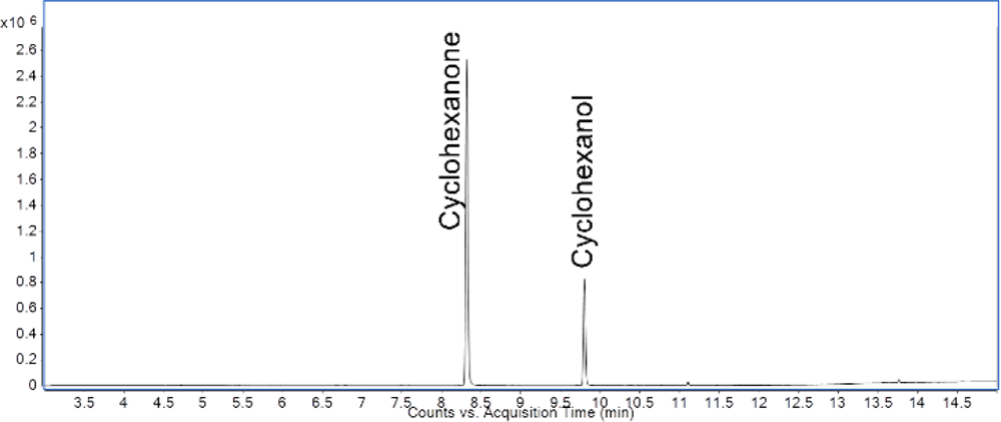
Headspace GC/MS chromatogram of 200 ppm standards of cyclohexanone and cyclohexanol.

A 500 μL aliquot of the headspace gas from each headspace vial was injected into the split/splitless inlet (20:1 split ratio) onto an Agilent 7890 GC system, fitted with a DB5-MS GC column 30 m × 0.25 mm, 0.25 μm (Agilent Technologies, Santa Clara, CA). The oven was initially held at 40 °C for 1 min before being ramped from 10 °C for 1 min to 120 °C followed by further ramping from 40 to 250 °C where it was held for the remainder of the 15 min run time. MS was operated in selected ion monitoring mode with an electron impact source (70 eV) using *m/z* 55 and 98 for cyclohexanone and *m/z* 57 and 82 for cyclohexanol. The source and quadrupole temperatures were 230 and 150 °C, respectively. Figure 5 shows an example chromatogram.

### Headspace-SIFT-MS Analysis

**Figure 6:**
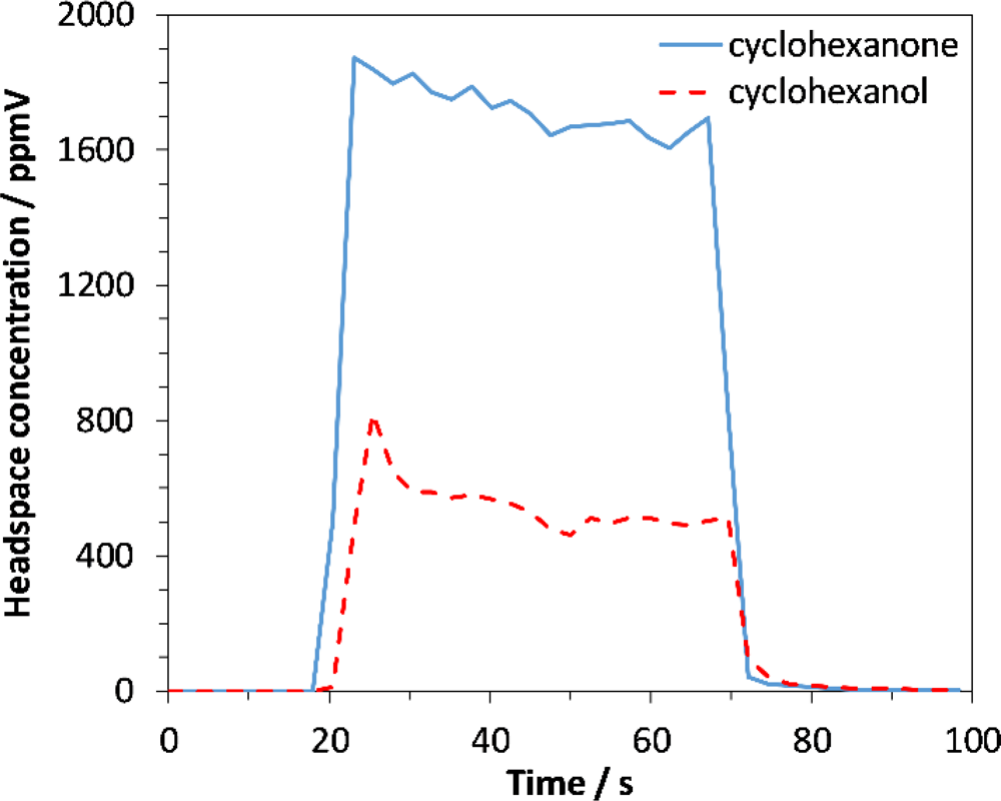
SIFT-MS time series of a 200 ppm cyclohexanone/cyclohexanol standard; trace shows a period of no sample addition followed by the injection period, and then the injection end.

A 2500 μL aliquot of the headspace was injected into SIFT-MS at a speed of 50 μL s^–1^. Cyclohexanone^19^ was quantified using reagent ion H_3_O^+^ with product ions *m/z* 99 and 117 (the latter ion being a secondary water adduct) and reagent ion NO^+^ with product ions *m/z* 98 and 128 (the latter being an adduct of NO^+^). Cyclohexanol^20^ used the H_3_O^+^ reagent ion with product ion *m/z* 83 and NO^+^ reagent ion with product ions *m/z* 99 and 117 (water adduct). Figure 6 shows an example of a time series of automated SIFT-MS injection of a standard.
